# Behçet’s Disease—Do Microbiomes and Genetics Collaborate in Pathogenesis?

**DOI:** 10.3389/fimmu.2021.648341

**Published:** 2021-05-21

**Authors:** Nafeesa Mehmood, Liying Low, Graham R. Wallace

**Affiliations:** Institute of Inflammation and Ageing, College of Medical and Dental Sciences, University of Birmingham, Birmingham, United Kingdom

**Keywords:** Behçet’s disease, microbiome, genetic polymorphism, gut, butyrate

## Abstract

Behçet’s disease (BD) is a multisystem autoinflammatory condition characterized by mucosal ulceration, breakdown of immune privilege sites and vasculitis. A genetic basis for BD has been described in genome-wide and validation studies. Similarly, dysbiosis of oral and gut microbiomes have been associated with BD. This review will describe links between genetic polymorphisms in genes encoding molecules involved in gut biology and changes seen in microbiome studies. A potential decrease in bacterial species producing short chain fatty acids linked to mutations in genes involved in their production suggests a potential therapy for BD.

## Introduction

Behçet’s Disease (BD) is a multisystem autoinflammatory disorder characterized by inflammation at mucosal surfaces and break down of barriers at privileged sites including the eye, brain and joint (1). Many of the mucosal surfaces involved in BD host a community of microorganisms, and collectively their genetic content is known as the microbiome. The largest and most diverse of these microbial communities reside in the gut and have been shown to be important in the development and regulation of the immune system (2). Changes in the gut microbiome, also known as dysbiosis, have been associated with many human diseases including chronic kidney disease, diabetes, multiple sclerosis (3–5). The gut microbiome has also been shown to modulate the efficacy of immunotherapies in cancer patients (6, 7).

Since the first description of the disease triad in the 1930s, infectious microbial agents have always been speculated to be involved in the pathogenesis of BD, however, so far, no single pathogenic etiology has been found. Recent studies have investigated the association between the composition of the microbiomes and BD, which may provide a useful insight to the role of the microbiome in the disease process.

### Gut Microbiome in Behçet’s Disease

The gut microbiome profile in BD was first reported by Consolandi and associates, whereby they compared the fecal microbiota of 22 patients with 16 age-matched cohabitating individuals. Recruiting healthy controls who lived in the same house as the BD patients is of particular importance as it is known that several factors contribute to variations in gut microbiota composition, including environmental factors, geographical location, diet, age and host genetics (6). There was significant reduction in the alpha diversity and depletion of butyrate-producing bacteria, such as *Roseburia* and *Subdoligranulum* genera, in the gut microbiome of BD patients compared to healthy controls (**Table 1**). The short chain fatty acid (SCFA) profile, by chromatography-mass spectrometry (GC-MS), revealed a significant reduction of butyrate levels in fecal samples of BD patients. Butyrate is important in regulating the host immune system and is responsible for inhibiting intestinal pro-inflammatory cytokines and promoting T cell differentiation into a T regulatory cell (Treg) lineage. Thus, it can be hypothesized that the dysbiotic microbiota seen in BD patients could have an important role in the pathogenesis of BD, whereby a reduction in butyrate causes downstream effects of promoting immune-pathological T-effector responses, leading to gut inflammation (18). Interestingly, a similar pattern is seen in patients with inflammatory bowel disease (IBD) and oral administration of butyrate in experimental IBD mice, has shown to enhance Treg differentiation and reduce colitis (8, 19).

**Table 1:** Summary of microbiome dysbiosis in Behçet’s Disease patients compared to healthy controls or disease controls.

Author	MicrobiomeBody Site (Country)	Increased bacterial abundance in BD patients	Reduced bacterial abundance in BD patients
Consolandi, C., et al. (7)	**Gut** (Italy) P=22 HC=16		***Roseburia (Clostridium* genus*)*** ***Subdoligranulum (Clostridium* genus*)***
Shimizu, J., et al. (8)	**Gut** (Japan) P=12 HC=12	*Bifidobacterium* *Eggerthella*	*Megamonas* *Prevotella*
Shimizu, J., et al. (9)	**Gut** (Japan) P=13 HC=27	*Eggerthella lenta* *Acidaminococcus* spp. *Lactobacillus* spp. *Bifidobacterium bifidum* *Streptococus* spp.	*Megamonas hypermegale* ***Butyrivibrio* spp.** *Filifactor* spp.
Ye, Z., et al. (10)	**Gut** (active ocular BD patients from China) P=24 HC=52	*Bilophila* spp. *(Sulfate-reducing)* *Parabacteroides* spp. *Paraprevotella* spp.	***Clostridium* spp. *(Butyrate-producing bacteria)*** *Methanoculleus* spp. *Methanomethylophilus* spp.
Oezguen N., et al. (11)	**Gut** (neuro-BD patients Turkey)	*Parabacteroides* *Clostridiales* *Gemminger* *Butyricimonas* *Actinobacteria*	*Vampirovibrio* *Unclassified Lachnospiraceae*
Tecer D, et al. (12)	**Gut** (BD-uveitis, FMF, CD patients Turkey) BD=7 FMF=12 CD=9 HC=16	*Veionellaceae* *Succinivibrionaceae* *Succinivibrio* *Mitsuokella*	
Yaser Bilge et al. (13)	**Gut** (Turkey) P=27 HC=10	*Actinomyces* *Libanicoccus* *Collinsella* *Eggerthella* *Enetrohabdus* *Catenibacterium* *Enterobacter*	*Bacteroides* *Cricetibacter* *Alistipes* *Lachnospira* *Dielma* ***Akkermansia*** *Sutterella* *Anaerofilum* *Ruminococcease Acetanaerobacterium Copropaacter*
Van der Houwen J. et al. (14)	**Gut** (Italy and Netherlands; IgA coated bacteria- Netherlands only) BD Dutch=19 HC Dutch=17 BD Italy=13 HC Italy=15	*Bifidobacterium* spp., *Dorea* spp., *Ruminoccoccus bromei* spp. (all IgA coating in Netherlands cohort)	*Barnesiellaceae* *Lachnospira* *(Italy only)* *Erysipelotrichaceae* spp. (IgA coating in Netherlands cohort)
Coit, P., et al. (15)	**Oral** (Turkey) P=31 HC=15	*Haemophilus parainfluenza*	*Alloprevotella rava* *Leptotrichia*
Seoudi, N., et al. (16)	**Oral** (UK) BD=54 RAS=8 HC=25	*Rothia denticariosa* *Streptococcus salivarius* *Streptococcus sanguinis*	*Neisseria* *Veillonella*
Balt J. et al. (17)	**Oral** (Mongolia) P=47 HC=48		***Akkermansia***

The table shows the relative abundance of microbial species in the microbiota (gut and oral microbiota) of BD patients when compared to HCs. **Bold** = species or genera linked to T regulatory cells. Abbreviations: BD, Behçet’s Disease; HC, healthy control; FMF, Familial Mediterranean Fever; CD, Chron’s Disease CD; MS, Multiple Sclerosis, RAS, Recurrent aphthous ulcer.

In a pilot 16S gut microbiome study of 12 Japanese patients with BD and 12 healthy controls (HC), Shimizu and colleagues reported a significant increase in the relative abundance of *Bifidobacterium* and *Eggerthella*, and decrease in the relative abundance of *Megamonas* and *Prevotella* in BD patients compared with controls, however there was correlation between the bacterial taxa and clinical manifestation of BD (9). (**Table 1**) Fecal soluble immunoglobulin A (sIgA) concentrations were also significantly increased in BD patients but did not correlate with any specific bacterial species (9). In a follow up study analysis of the taxonomic and predicted metagenome functional composition in the fecal sample of 13 Japanese patients with BD and 27 HC, the relative abundance of *Lactobacillus, Bifidobacterium* and *Streptococcus* spp. was significantly increased in patients with BD compared to HC, whilst *Megamonas hypermegale* and *Butyrivibrio* species were less abundant, which correlated with the previous study (**Table 1**). The latter species were suggested to produce SCFA, particularly butyrate and propionate, in the human intestine. Such a reduction in butyrate-producing-SCFA may be responsible for the skewed T cell differentiation, ie. an increase in Th17 with a concomitant reduction in Treg cells. Functional annotation analysis found links to the pentose phosphate pathway and inosine monophosphate biosynthesis, suggesting that changes in the microbiome could affect metabolic processes in patients with BD. Nevertheless, intestinal microbiome interactions with the immune response are complex and SCFA depletion may only be one of many contributing factors to BD pathogenesis (10).

Ye et al. compared the DNA extracted from fecal samples of 24 patients with active BD, who had only received topical steroids, and 52 gender, body mass index (BMI) and aged-matched healthy controls (HC) (11). Metagenomic analysis revealed significant differences in gut microbial composition between the two cohorts with sulfate-reducing bacteria (SRB) and opportunistic pathogens significantly enriched, and methanogens, *Methanoculleus* spp. and *methanomethylophilus* ssp. and butyrate-producing bacteria, *Clostridium* spp. were reduced in BD patients compared to controls. SRB are pro-inflammatory bacteria that can inhibit butyrate. The shortage of butyrate and methane, combined with an abundance of hydrogen sulphide, creates an atypical environment that may contribute to intestinal epithelial barrier damage. This may allow for subsequent invasion by Th1 and Th17 cells, reduction in Tregs, thus triggering downstream inflammatory processes within the gut in patients with BD. To address this Ye *et al.* used the animal model of experimental autoimmune uveitis in B10RIII mice. Mice were treated with antibiotics (ampicillin, neomycin, metronidazole and vancomycin) for 3 weeks, prior to daily transplantation of pooled fecal suspension either from patients with active BD or from healthy controls. After one week of daily fecal transplants, mice were immunized with an interphotoreceptor retinoid binding protein peptide (161-180) to induce EAU and the severity of uveitis was reported on day 14 following immunization. The results showed that severe uveitis (corneal oedema, posterior synechiae and infiltrates of inflammatory cells in retina, choroid and vitreous humor) was observed in the active BD-recipient group, whilst the HC-recipient group displayed a slight intraocular inflammatory reaction with very few inflammatory cells in the vitreous cavity, indicating that the components within the fecal samples from BD patients exacerbated the development of intraocular inflammation in these mice (11). This supports the hypothesis that gut microbiome composition may contribute to BD pathogenesis and severity.

In a novel study the gut microbiome composition of Turkish patients with neuro-BD was compared to patients with multiple sclerosis, an autoimmune condition, and to healthy controls. 16S rRNA gene sequencing revealed that neuro-BD patients had an increased relative abundance of *Parabacteroides, Geminger* and *Prevotella*, whilst *Vampirovibrio* and *Lachnospiraceae* were decreased (**Table 1**), although the differences did not reach statistical significance, probably due to the small cohort sizes. However, it is of interest that two neurological conditions were linked to different changes in the intestinal microbiota. Whether this is due to the different disease processes or treatment regimens for participants, as those with neuro-BD had commenced azathioprine (with or without colchicine treatment), whilst MS patients had commenced immunomodulatory drug treatment (drug treatment not specified), will have to be investigated further (12).

The gut microbiome was compared in patients with BD uveitis to patients with Familial Mediterranean Fever (FMF) and Crohn’s Disease (CD), both which have autoinflammatory processes, as well as healthy controls. In total seven patients with BD, 12 patients with FMF, 9 patients with CD and 16 healthy controls (HC) were analyzed by 16S rRNA gene sequencing. The results showed that *Veionellaceae, Succinivibrionaceae* families and *Succinivibrio* and *Mitsuokella* genera were enriched in patients with BD compared to the other groups. The relevance to uveitis is not yet elucidated (15).

### Oral Microbiome in Behçets Disease

Oral ulceration is an important manifestation of BD and maintains a specific microbiome. To investigate the involvement of the oral microbiome in BD pathogenesis, Coit et al. collected stimulated saliva samples from 31 BD patients and 15 HCs, after 5 minutes of paraffin chewing, between 9-11am to address circadian rhythm of saliva secretion. Microbial DNA was isolated and sequencing revealed a significant difference in bacterial communities between the two cohorts. *Haemophilus parainfluenzae* and *Alloprevotella* were increased in BD patients, whilst *Leptotrichia* and *Clostridiales* were decreased compared to control samples. *H. parainfluenzae*, a gram-negative bacterium, is commonly linked to endocarditis, meningitis, septicaemia and gingival inflammation. While use of immunosuppressants (cyclosporin, azathioprine and prednisolone) had an impact on abundances of bacteria, there was no differences in bacterial communities between drug treatments (16).

In a second study, the oral microbiome profiles of BD patients (active vs. inactive disease) were compared to patients with recurrent aphthous ulcers (RAS), used as disease control, and healthy individuals. In total, unstimulated saliva samples were collected from 54 patients with BD, 8 patients with RAS and 25 HC together with mucosal samples from some patients. The results showed differences between the cohorts, with *Neisseria* more commonly isolated from HC compared to BD patients, whereas *Staphylococcus salivarius* and *Streptococcus sanguinis* were more abundant in BD patients with active oral disease when compared to those with inactive disease, patients with RAS and HC. *Candida albicans* was more frequently isolated from salivary samples taken from orally inactive BD patients when compared to HCs and RAS, however this could be attributed to steroid treatment. Samples taken from ulcerated and non-ulcerated mucosa of active BD patients was compared to those from HCs and RAS and revealed *Rothia dentocariosa* as the abundant organism in non-ulcerated mucosa of active BD patients, with a trend toward *Rothia mucilaginosa* from the ulcer sites of active BD patients (17).

In a recent study the oral microbiome in Mongolian patients with BD was compared to healthy controls. Saliva samples were obtained from 47 patients and 48 healthy individuals and the V3-V4 region of 16S RNA was amplified. Specifically, Prevotella, Veillonella, Streptococcus, and Haemophilus were the most abundant species in all samples However, three genera including, Akkermansia were significantly lower in patients compared to controls (20).

### Gut and Oral Microbiomes in BD—Summary

These studies support a possible role for gut or oral dysbiosis in BD. Common features such as the reduction in butyrate producing species can be found in most studies, although different bacterial species are reported in different studies. Despite the evidence of microbial dysbiosis in BD patients, it is difficult to ascertain whether such differences are causative or reactive in BD. This is due to many confounding factors that could affect the microbiome including the type and duration of drug treatment, sampling and sequencing methodology, or host genetics. This could also be linked to the different manifestations of BD, which could only be addressed through large longitudinal studies.

To distinguish disease-associated determinants from country and methodology, the gut microbiomes of patients with BD from the Netherlands and Italy were analyzed by the same method and compared to each other and healthy controls. In both cohorts, alpha diversity were similar to controls. Beta-diversity analysis did not show clustering based on disease, however, there was segregation by country of origin. The Italian cohort showed a significant decrease in *Barnesiellaceae* and *Lachnospira* genera, which in the Netherlands cohort was only a trend. IgA coated of intestinal bacteria analyzed by RNA sequencing can identify distinct disease-associated species in fecal samples. Increased IgA coating of *Bifidobacterium* spp., *Dorea* spp., and *Ruminoccoccus bromei* spp. and reduced presence of IgA-coated *Erysipelotrichaceae* spp. in the Dutch BD cohort compared to healthy controls, although this was not analyzed in the Italian cohort (14). These data support the requirement for more, larger studies to determine the geographical and environmental changes compared to disease-induced changes in the BD gut microbiome.

Behçet’s Disease often involves variable disease manifestations at different body sites, such as the oral, skin, eyes, gastrointestinal, or nervous system, over time. To identify the clinical clusters of BD, Soejima and associates investigated the clinical phenotype of 657 patients with BD from the Yokohama City Hospital database and 6754 patients with BD from the Japanese Ministry of Health, Labour and Welfare database. They identified that gastrointestinal BD was associated with vascular but not eye involvement, while a second cluster had high incidence of eye involvement but no vascular or neurological involvement (13). Chronological analyses showed expansion of the gastrointestinal cluster in BD patients over 27 years in a relatively stable genetic cohort in Japan, indicating that environmental factors might be involved in the disease manifestation of BD.

Changes in the gut microbiome could drive the clinical phenotype in BD. To answer this, Yasar Bilge and associates compared the gut microbiome of 27 patients with BD (ocular, mucocutaneous, and vascular involvement), and 10 age- and sex-matched healthy controls. Linear discriminant analysis of this small patient cohort revealed a difference in the bacterial genera according to the manifestations of BD: *Lachnospiraceae NK4A136* enriched in BD patients with eye involvement, *Dialister*, *Intestinomonas*, and *Marvinbryantia* enriched in BD patients with predominant mucocutaneous disease, *Gemella* in vascular BD. However, larger studies and functional relevance of such associations should be investigated (21).

This data, if validated in other patient cohorts, supports the stratification of individuals in relation to their gut microbiome profile (i.e. to determine if specific bacterial species are associated with specific manifestations or whether general alterations to the microbiome influences all manifestations). In either process, factors that may potentially influence the gut microbiome, such as diet, can be addressed by the potential of re-establishing a more balanced, symbiotic relationship might prove to be important in reducing the severity of active BD phases. To test this concept a randomized controlled trial the Modulation of Gut Microbiota through Nutritional Invention in Behçet’s Syndrome Patients (MAMBA) study, will recruit 90 patients with BD, to investigate the impact of three dietary interventions, a lacto-ovo-vegetarian diet, a Mediterranean diet, or a Mediterranean diet supplemented with butyrate on the severity of clinical manifestations of BD (22).

There is increasing evidence that the host genetic background shapes the gut microbiome profile (23, 24).

### Link Between Gut Microbiome and Genetics in BD

There are more than 20 genetic susceptibility loci that meet the threshold for genome-wide level of significance in patients with Behçet’s disease and several other loci that have been associated with BD (25). To address the potential role of genetics in BD gut dysbiosis we have selected a potential pathway associated with production of SCFA in the gut and mutations in the genes involved. Activation of Toll-like receptors on systemic dendritic cells induces the release of IL-23, which activates innate lymphoid cells to release IL-22 which leads to a rapid α (1,2)-fucosylation of small intestine epithelial cells *via* the release of fucosyltransferase 2 (FUT2). Fucosylated proteins enter into the lumen of the gut where they release fucose molecules which are metabolized by bacteria present. As stated, products from these bacteria include short chain fatty acids, including butyrate which supports T regulatory cells (26). This complex series of interactions involve several of the gene mutations in which have been associated with BD.

### Toll-Like Receptors

Toll-like receptors (TLR) are a family of transmembrane sensors for bacteria, fungi and viruses that are a vital component of innate immunity. The strongest associations between TLR and BD is in with TLR4 which recognizes bacterial lipopolysaccharide. Nine single nucleotide polymorphisms previously detected in TLR4 by direct sequencing were analyzed for an association with BD in Korean patients. The most frequent haplotype, TAGCGGTAA, was significantly increased in HLA-B*51-positive patients compared with healthy control participants and potential linked to joint disease (27). This was supported by targeted exonic resequencing of specific genes identified in the original GWAS, from 2,461 BD cases compared with 2,458 controls where a SNP in TLR4 was significantly associated with BD (28). In buccal lesions from patients with BD, TLR expression was increased compared with healthy controls, however, a similar increase was seen in lesion tissue from patients with lichen planus or gingivitis, suggesting that this was a generalized inflammatory response as opposed to a BD-specific response. In these patients no significant associations were seen with TLR4, but a gain of function mutation in the TLR adaptor molecule, TIR-adaptor protein (TIRAP) was significantly associated with BD in UK, but not Middle Eastern, patients (29). This mutation is associated with an more potent cytokine response to TLR stimulation.

In a study of 400 patients with ocular BD compared to 600 healthy Han Chinese controls frequencies of genotypes of TLR2 rs2289318 and rs3804099 were significantly higher in ocular BD patients compared with controls. A validation cohort of 438 patients with BD uveitis and 1000 healthy controls confirmed the associations. TLR2 mRNA expression in PBMC was increased in healthy carriers of the certain genotypes, although this did not affect the release of TNF-α, IL-6, IL-10, and IL-1β could be detected (30).

In Tunisians patients with Behcet’s Disease expression of SNP in TLR9 (1486 T/C) was different between BD patients and healthy controls but did not reach statistical significance (31). In Chinese patients with BD TLR7 had an increased copy number and increased expression in PBMC compared to healthy controls (32).

These data support a role for bacterial sensing in BD.

### Interleukin-23

Two major GWAS studies in Turkish and Japanese patients with BD identified SNP at the IL23R-IL12RB2 locus (rs924080 and rs12119179) respectively. Meta-analysis of both data sets further identified rs1495965 as associated with BD (33, 34). These associations have been supported in several other studies in different patient cohorts (35–39). These mutations have been linked to enhanced expression of IL-23R and potential upregulation of the IL-17 pathway.

Lymphocytes isolated from the intestinal mucosa of 8 patients with intestinal symptoms, that had started within 6 months of collection, were cloned and analyzed for surface phenotype and cytokine production. Results showed more CD4^+^ clones compared to CD8^+^ with the majority of both showing Th1 profile secreting IFN-γ and TNF-α, with a substantial population produced both IFN-γ and IL-17 (Th1/Th17 profile). Most of the T-cell clones showed perforin-mediated cytotoxicity and Fas–Fas Ligand-mediated pro-apoptotic activity. Therefore, in the early stages of intestinal BD both Th1 cytokines, IL-17 and cytotoxicity are involved in inflammation of the mucosal tissue. This data would support the use of anti-TNF treatment and potentially anti-IL-17 in controlling intestinal BD (40).

In support of treatment altering the Th17 pathway, twenty-seven patients who received interferon alfa-2a for active BD uveitis and Treg and Th17 cells were assessed. Treg, Th17 cell frequencies and Th17 RORγt expression were significantly elevated, although IL-10 concentration in Treg culture supernatants was significantly lower with cells from BD patients compared to controls. IFN alfa-2a treatment led to clinical remission in 70% of patients which correlated with a significant reversal in Treg and Th17 cell frequencies, Treg IL-10 and Th17 cytokine production (41).

### Interleukin-22

IL-22 is a member of the IL-10 cytokine family that is involved in inflammatory processes. Th22-type T cell clones from ocular samples taken from Behçet’s disease patients with active uveitis, produced IL-22 and TNF-a but not IFN-γ or IL-17. IL-22 can affect epithelial and stromal cells and has been involved the pathogenesis of inflammatory skin disorders, such as psoriasis, atopic eczema, and allergic contact dermatitis (42). Healthy IL-22-deficient mice had altered microbiome, with decreased abundance of *Lactobacillus*, and increased levels of others. Induction of experimental colitis caused more sever disease in deficient mice compared to controls. When the microbiome from IL-22 deficient mice was transmitted to wild-type animals along this led to an increased susceptibility to colitis, suggesting an important role for IL-22 the balance between immunity and colonic microbiota (43). Therefore, although no polymorphisms in *IL22* have been reported in BD, a potential role in pathogenesis is likely.

### Fucosyltransferase-2

Genome-wide array analysis of Iranian patients with BD and controls identified an association with five coding polymorphisms of the gene encoding fucosyltransferase -2 (FUT2), including the rs601338 nonsense variant which, in Caucasians, determines the secretion of the H antigen (precursor of the ABO blood group antigens) in body fluids and on the intestinal mucosa. These results were validated by comparison with GWAS analysis of Turkish patients with BD. A non-secretor phenotype is suggested to link with intestinal glycosylation (44). The association with FUT2 polymorphisms and BD was supported in an larger study of three populations. The rs601338 A allele, in Turkish and Iranian patients and the rs1047781 T allele in Japanese patients for which homozygosity leads to an ABO non-secretor phenotype were confirmed in this study. These non-secretor genotypes are also associated with Crohn’s disease risk (45).

The process by which FUT2 polymorphisms influence the gut microbiome is not yet clear. Secretor mothers produce milk containing α1-2 fucosylated human milk oligosaccharides and several strains of bacteria in the infant gut have the capacity to utilize these oligosaccharides (46). In vaginally born infants, the mother’s secretor status had no effect on the infants’ microbiome. By comparison, in children delivered by caesarean section several differences were seen in infants from secretor mothers compared to non-secretor mothers. In particular, *Bifidobacteria* were depleted and Enterococci increased among the caesarean-born infants of non-secretor mothers. *Akkermansia* was increased in infants of secretor mothers. In a study of 14 non-secretor (FUT2 rs601338 genotype AA) and 57 secretor (genotypes GG and AG) adult individuals of western European descent, the gut microbiota was analyzed. Results showed a several differences between the two groups with nonsecretors presenting lower species richness than secretors and different enterotypes in the two groups (46). This data supports the concept that FUT2 polymorphism can influence the composition of the gut microbiota and variation between individuals (47). By comparison, the association of rs601338 and intestinal microbiota composition was analyzed in 1,190 healthy individuals, and the results showed no association of the FUT2 genotype, secretor status and microbial alpha diversity, microbial composition or inferred microbial function after correction for multiple testing (48).

Non-secretors, who are homozygous for the loss-of-function alleles of FUT2 gene, have increased susceptibility to Crohn’s disease (CD). From 39 healthy individuals, 75 endoscopic lavage samples were profiled for microbiome, proteome and metabolome composition based on FUT2 genotype. Metagenomic analysis revealed differences in energy metabolism in the microbiome of non-secretor and heterozygote individuals, in particular enrichment of carbohydrate, vitamin and lipid metabolism, and depletion of amino-acid biosynthesis and metabolism. Metabolomic analysis of human specimens showed changes of many metabolites related to the different genotype groups. These results support compositional and functional differences in the microbiota of non-secretors that may be relevant for CD susceptibility (49).

Taken together polymorphisms in all genes encoding proteins in the pathway TLR-IL-23-IL-22- FUT2 have all been reported in patients with BD. Systemic activation of this pathway could lead to a reduction in beneficial SCFA either by increased targeting of SCFA-producing species or *via* reduced fucosylation and SCFA generation. This is one potential pathway initiated by systemic activation of tissue dendritic cells. The best direct evidence of cause and effect remains the induction of uveitis in B10RIII mice following transplantation of fecal material from patients with active BD (11). However, it is not known what induced the changes in the microbiome of these patients nor what processes drove ocular inflammation even in this genetically susceptible mouse model.

### Summary

A possible link between gut and oral microbiome dysbiosis is supported by several studies and the involvement of the FUT2 pathway and gene polymorphisms in molecules involved may be relevant. However, there are several aspects that must be addressed. First, that different bacterial species and genera have been identified in the different studies. While there may be a commonality in decreased abundance of species linked to short chain fatty acid regulation that could link the studies more work needs to be done in larger cohorts with the same collection and preparation procedures. Second, certain demographic analysis does not support a link between manifestations of BD form example ocular involvement not associated with intestinal BD (13). Thirdly, many patients with BD do not have intestinal-BD, therefore there is no direct association between gut inflammation, the microbiome and other manifestations of BD. Fourth, several other sites affected by BD including oral and genital mucosa and skin harbor their own microbiome, which will need to be analyzed for association with manifestations of BD. Fifth, there is a significant non-genetic component in Behçet’s Disease. As stated, the MAMBA study (22) will address dietary influences on the gut microbiome. Recently the numbers of new patients being diagnosed with BD in Japan and Korea have been steadily decreasing (50, 51). Whether such changes involve factors such as a move to a more westernized diet would be interesting to address. Effects of different treatment regimes on the gut microbiome should be investigated. Finally, there are other gene loci that have been associated with BD that could impact on the composition of or the response to the gut microbiome, including HLA-B*51 and *IL-10* and cells such as natural killer cells which can produce IL-10 when stimulated. Moreover, IL-10 production by Treg has been linked to *Akkermansia* which is altered in several studies.

**Figure 1:**
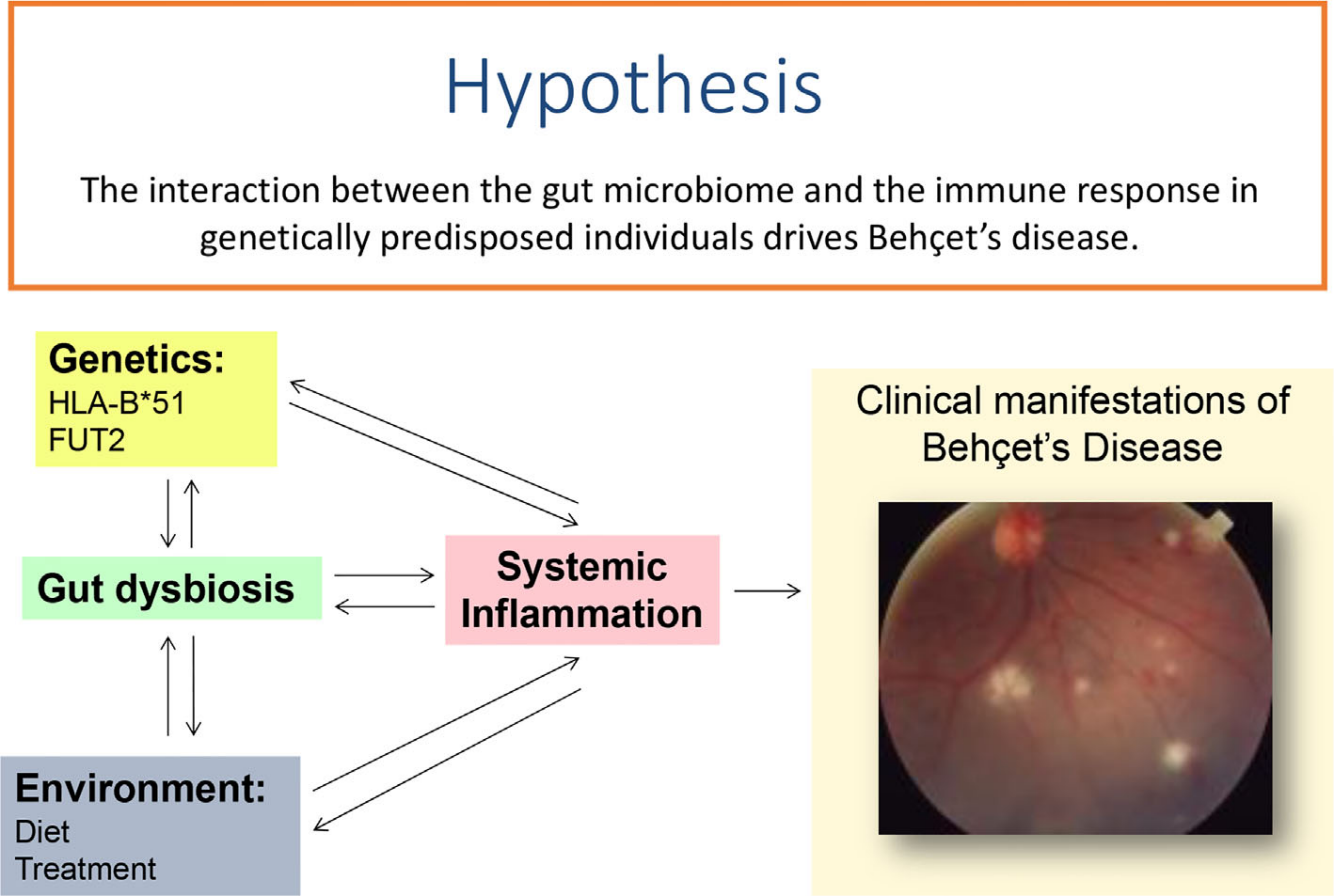
Systemic inflammation leading to clinical features in Behçet’s disease may either be initiated by of causing changes in the gut microbiome in patients who are genetically predisposed to the condition.

In conclusion, while it is clear that dysbiosis of the gut microbiome is associated with Behçet’s disease and a role for known genetic mutations can be linked to these changes (**Figure 1**), further research is required to understand the combinations involved in the manifestations of this complex disease.

## Author Contributions 

All authors contributed equally to this manuscript. All authors contributed to the article and approved the submitted version.

## Funding

Liying Low is funded by a Fight for Sight Clinical Research Fellowship (Ref 1840/1841).

## Conflict of Interest

The authors declare that the research was conducted in the absence of any commercial or financial relationships that could be construed as a potential conflict of interest.
